# Gene-based analysis of rare and common variants to determine association with blood pressure

**DOI:** 10.1186/1753-6561-8-S1-S46

**Published:** 2014-06-17

**Authors:** Xiaofeng Liu, Joseph Beyene

**Affiliations:** 1 Population Genomic Program, Department of Clinical Epidemiology & Biostatistics, McMaster University, 1280 Main Street West, Hamilton, Ontario L8S4K1, Canada

## Abstract

Systolic blood pressure and diastolic blood pressure are known risk factors for cardiovascular diseases and understanding their genetic basis will have important public health implications. For rare variants, it is extremely challenging to make statistical inference for single-maker tests. Therefore, joint analysis of a set of variants has been proposed. In this paper, we applied recently proposed methods "test for testing the effect of an optimally weighted combination of variants" and "variable weight-TOW" to determine genetic regions that are associated with blood pressure. Then least absolute shrinkage and selection operator, as well as sparse partial least square methods, were used to identify significant markers within a gene or in intergenic regions. We investigated the effect of rare variants and common variants, and their combined effect.

## Background

It is well known that high blood pressure is an important risk factor for cardiovascular diseases. Elevated blood pressure is a complicated trait that affects more than 30% of the adult population [1,2]. An increase in systolic and diastolic blood pressure has a continuous impact on the risk of cardiovascular diseases. Globally, every year, high blood pressure contributes to approximately 13.5% of premature deaths, 54% of stroke, and 47% of ischemic heart disease [1,3]. Genetic heritance is one of the major risk factors for hypertension. For complex diseases, the common disease-common variant (CD-CV) hypothesis that underpins genome-wide association studies (GWAS) has led to the identification of several novel susceptibility loci. However, a majority of the heritability is unexplained. It has been pointed out that the GWAS-identified variants can only explain a small portion of the heritability; therefore, exploration is still needed to unveil the undiscovered variants [12]. Recently, arguments have been put forward against CD-CV, and common disease-rare variants (CD-RV) as an alternative has been proposed. It is based on the assumption that the etiology for common diseases is caused by the cumulative effect of multiple rare variants [4,5]. Nevertheless, another merging hypothesis states that common diseases are caused by the combination of common and rare variants [6-8].

In this paper, we focused on identifying whether a gene is associated with blood pressure. We applied recently proposed tests called "test for testing the effect of an optimally weighted combination of variants (TOW)" and "variable weight-TOW (VW-TOW)" [9] to determine significant genetic regions. Our interest also lies on identifying the associated variants for regions that are found significantly associated by applying sparse methods Lasso and SPLS [10,11].

## Methods

### Data

Both the real and simulated data that were made available for Genetic Analysis Workshop 18 (GAW18) were used. We focused on the genotype data on chromosome 3 for unrelated individuals. The baseline data for the covariates and the phenotypes were considered. We considered the first time point of systolic blood pressure (SBP) and diastolic blood pressure (DBP) as the traits. We also used a composite of the 2 phenotypes called the mean arterial pressure, which is defined as (2/3)*DBP + (1/3)*SBP. For the genotype data, we mapped single-nucleotide polymorphisms (SNPs) to the genes; the remaining SNPs that do not belong to any genes, were grouped as intergenic regions. A total of 2286 regions (consisting of 1224 genes and 1062 intergenic regions) that include all the SNPs were defined. The regions were further divided into "rare" or "common" based on minor allele frequency (MAF) threshold of 0.01.

### Association tests

TOW and VW-TOW are recently proposed methods that allow covariates and account for direction effects for causal variants. Let $Z_{i}={\left(z_{i1},\ldots ,z_{ip}\right)}^{T}$, $X_{i}={\left(x_{i1},\ldots ,x_{iM}\right)}^{T}$ and $y_{i}$ be the covariates, genotype (coded 0, 1, 2) and phenotype for the *i*^th ^individual, where *p *and *M *denote number of covariates and variants, respectively. The effects of the covariates on $y_{i}$ and $x_{im}$ are adjusted by the residuals of the following linear models

(1) $$y_{i}=\alpha_{0}+\alpha_{1}z_{i1}+\ldots +\alpha_{p}z_{ip}+\varepsilon_{i}$$

and

(2) $$x_{im}=\alpha_{0m}+\alpha_{1m}z_{i1}+\ldots +\alpha_{pm}z_{ip}+\tau_{im}.$$

The methods are based on the optimal weighting scheme, which is defined as $w_{m}^{o}=\frac{\sum_{i=1}^{n}\left({ỹ}_{i}-ỹ\right)\left({\tilde{x}}_{im}-{\tilde{x}}_{m}\right)}{\sum_{i=1}^{n}{\left({\tilde{x}}_{im}-{\tilde{x}}_{m}\right)}^{2}}$, where ${ỹ}_{i}$ and ${\tilde{x}}_{im}$ denote the residuals from equations (1) and (2) for the *i*^th ^individual respectively. Let $x_{i}^{o}=\overset{M}{\underset{m=1}{\sum }}w_{m}^{o}{\tilde{x}}_{im}$. The test statistics for TOW is defined as $T_{TOW}=\overset{n}{\underset{i=1}{\sum }}\left({ỹ}_{i}-ỹ\right)\left(x_{i}^{o}-x^{o}\right)$. For VW-TOW, let $T_{r}$ and $T_{c}$ denote the test statistics of TOW for rare and common variants, $T_{\lambda }=\lambda \frac{T_{r}}{\sqrt{var\left(T_{r}\right)}}+\left(1-\lambda \right)\frac{T_{c}}{\sqrt{var\left(T_{c}\right)}}$ and $p_{\lambda }$ be the *p *value of $T_{\lambda }$. The test statistics for VW-TOW is defined as $T_{VW-TOW}={\text{min}}_{0\leq \lambda \leq 1}p_{\lambda }={\text{min}}_{0\leq \text{k}\leq \text{K}}p_{\lambda_{k}}$, where $\lambda_{k}=\text{k}/\text{K}$ for k=0,1,⋯,K. The *p *values are evaluated by permutation.

After identifying the significant genomic regions, we further investigated the SNPs that have important contribution to the phenotypes for the significant regions by variable selection methods Lasso and SPLS, which are available in the R package: "RV tests." Because this package does not allow covariates, we adjusted the effect of environmental factors using the linear model shown in equation (1). Instead of the observed trait, the residuals from the linear model are treated as the phenotype.

### A summary of the steps we followed for real data

Step 1: Map the SNPs to gene and intergenic regions based on the annotation file refGene.txt.gz (available from http://hgdownload.cse.ucsc.edu/). Then the genes or intergenic regions were further divided into subregions ("rare" vs. "common") based on a threshold of MAF = 0.01.

Step 2: Extract the genotype, phenotype (baseline measures) and covariates (baseline measures) data for the unrelated individuals. Remove the participants that have missing variables in phenotype or covariates data.

Step 3: TOW and VW-TOW are applied to identify the regions that are associated with the traits.

Step 4: Apply Lasso and SPLS to the regions to discriminate the associated variants from noise (using the R package "RV tests").

## Results

### Real data

The sample used in our analysis is made up of 142 independent individuals. After removing missing variables, 129 subjects were analyzed. There are, in total, 1,215,296 markers on chromosome 3; approximately one-sixth of the markers were removed as a result of zero variation across the 129 independent samples.

**Figure 1:**
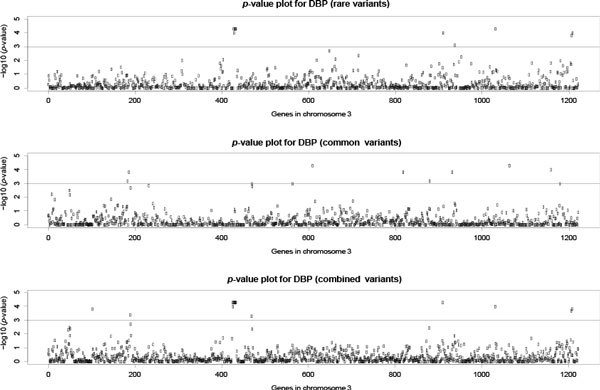
**p value plot for DBP (rare variants); p value plot for DBP (common variants); and p value plot for DBP (combined)**.

The association tests (TOW and VW-TOW) were applied to each genetic region for SBP, DBP, and mean arterial pressure (MAP) on chromosome 3. Both tests produce an empirical *p *value, based on 10,000 permutations for each region. Figure 1 displays the *p *value plot for DBP, where the x-axis denotes the position of the genes in original order on chromosome 3. The *p *values for intergenic regions are not included in Figure 1. By parallel comparison, we can see that effects of the genes are caused by the rare variants or the common variants. We note that there is a small cluster of genes that appear highly significant around the 440th region in the upper and lower plots.

After obtaining all the *p *values, regions that have strong association with the traits are picked according to the ranking of the *p *values. We decided to set the significant level threshold to be 0.001, so as to be more selective. Genes are only selected if they satisfy this criterion for the trait using both TOW and VW-TOW. Table 1 lists the regions that appear to be potentially important. For SBP, there are 3 genes; 2 genes with common variants only and 1 gene with rare variants only are highly associated with the trait. For MAP there are 4 genes when a combined analysis of "rare" and "common" variants is done, and 2 genes are significant with common variants only. For DBP, the number of significant regions is greater than the other 2 traits. For this trait, not only variants that belong to genes, but also variants in intergenic regions exhibit strong association.

**Table 1:** Common, rare and total number of variants identified by LASSO, SPLS and by both methods.

		Common variants					Rare variants					Combined variants			
						
Trait	Region	A	B	C	D	Region	A	B	C	D	Region	A	B	C	D
SBP	LAMP3	167	28	21	2	TP63	485	109	94	31	LAMP3	267	18	21	2

	LIMD1	371	20	39	15						LIMD1	538	88	39	18

											RAP2B	15	1	1	1

DBP	Inter-530	315	72	104	29	BAP1	24	7	8	7	BAP1	30	8	9	8

	CTDSPL	277	20	19	14	Inter-378	42	10	10	7	CCCDC66	373	42	57	20

	SLC25A36	88	7	6	6	Inter-896	23	4	8	4	Inter-376	10	1	2	1

						Inter-898	55	8	14	8	Inter-377	41	5	8	4

						CPB1	52	3	7	2	Inter-378	75	11	10	7

						DNAH1	211	40	41	16	Inter-896	47	4	8	4

						E1F5A2	34	11	5	5	Inter-982	206	27	2	2

						GLYCTK	13	4	5	4	CPB1	164	18	9	5

						NCBP2	24	2	2	2	DNAH1	326	114	43	31

						SEMA3G	30	7	8	5	EIF5A2	77	19	5	5

						TNNC1	7	3	4	3	GLYCTK	25	4	5	4

						WDR82	36	11	12	10	PHF7	40	3	14	3

											PIGZ	115	5	6	5

											PPM1M	12	2	2	2

											SEMA3G	42	7	8	5

											TNNC1	10	3	4	3

											TWF2	50	10	10	9

											WDR82	73	11	12	10

MAP	LAMP3	167	20	2	2						GP5	32	6	2	2

	LIMD1	371	10	51	5						LAMP3	267	3	2	2

											LIMD1	538	49	106	35

											RAP28	15	2	2	1

A, number of total variants; B, number of variants selected by LASSO; C, number of variants selected by SPLS; D, number of variants selected by both LASSO and SPLS; Inter-376, region between PPMIM & WDR82; Inter-377, region between WDR82 & GLYCTK; Inter-378, region between GLYCTK & DNAH1; Inter-530, region between NXPE3 & LOC152225; Inter-896, region between RPL22L1 & EIF5A2; Inter-898, region between SLC2A2 & TNIK; Inter-982, region between ST6GAL1 & RPL39L

As mentioned earlier, there is a cluster of regions (shown in Figure 1) that show strong significance for DBP. The region names are: TWF2, PPM1M, region between PPM1M and WDR82, WDR82, region between WDR82 and GLYC7K, GLYC7K, region between GLYC7K and DNAH1, BAP1, PHF7, SEMA3G, and TNNC1. The above regions all fall inside the physical location range of (52262625, 52488057).

Then variable selection methods Lasso and SPLS are applied to the regions that are picked at the gene (or region) level. Table 1 also summarizes the numbers of significant markers that were selected using these sparse methods. The number of selected markers can be varied with different choice of penalty parameter.

### Simulated data

In stage I, we focused on the top significant genes on chromosome 3, which are MAP4, FLNB, and ABTB1, with common and rare variants combined. We analyzed all 200 replicates with the target genes to assess the power of TOW and VW-TOW. MAP4 has large effect on both SBP and DBP, whereas FLNB and ABTB1 have small effects on SBP only. We adjusted the phenotypes by all the covariates at baseline. Table 2 reports the results. We can see that both methods have very poor power when the variants are all rare in the genes. Table 2 does show, however, that TOW has better performance than VW-TOW in most cases. MAP shows better power than the other 2 phenotypes. In the cases of small effect size, the power is very low for both TOW and VW-TOW.

**Table 2:** Power of TOW and VW-TOW to detect MAP4, FLNB, and ABTB1

		Power (MAP4)		Power (FLNB)		Power (ABTB1)	
		
	Traits	TOW	VW-TOW	TOW	VW-TOW	TOW	VW-TOW
Gene (combined)	SBP	0.32	0.265	0.02	0.015	0.095	0.07
	DBP	0.325	0.26				
	MAP	0.435	0.35				

Gene (rare)	SBP	0.035	0.03	0.005	0.01	0.095	0.075
	DBP	0.06	0.06				
	MAP	0.05	0.045				

Gene (common)	SBP	0.335	0.29	0.03	0.045	0.08	0.075
	DBP	0.345	0.245				
	MAP	0.445	0.33				

In stage II, we assessed the performance of Lasso and SPLS by analyzing all 200 replicates on MAP4 with all the variants. There are 6 target SNPs in MAP4, but 1 of the SNPs is removed because of monomorphism. The location numbers of the 5 SNPs are 48040283, 47957996, 47956424, 48040284, and 47913455. Both Lasso and SPLS are variable selection methods. With the careful selection of the penalty parameters for both methods, on average approximately 5 variants are selected with every replicate. Table 3 shows the results. We can see that using MAP as phenotype demonstrates higher power than using SBP or DBP. Lasso and SPLS have very poor power to detect 47956424 and 47913455.

**Table 3:** Power of Lasso and SPLS to select significant variants

		Power for each SNP(MAP4)				
	
	Traits	48040283	47957996	47956424	48040284	47913455
LASSO	SBP	0.53	0.255	0	0.38	0.065
	DBP	0.5	0.22	0	0.305	0.06
	MAP	0.705	0.265	0	0.52	0.075

SPLS	SBP	0.75	0.545	0.015	0.215	0.015
	DBP	0.66	0.43	0.01	0.185	0.03
	MAP	0.835	0.66	0.005	0.265	0.025

## Discussion

Most recently proposed methods assign large weights to rare variants and small weights to common variants, resulting in low power. On the other hand, TOW and VW-TOW assign corresponding weight, which can account for the direction effect, to individual variants. The methods outperform some currently popular methods, such as Combined Multivariate and Collapsing (CMC) and sequence kernel association test (SKAT), in various scenarios [9]. In addition, both TOW and VW-TOW can be modified to account for population stratification using principal component approach.

Overall, we were able to detect some significant genes based on association tests (TOW and VW-TOW) with SBP, DBP, and MAP. Although we used Lasso and SPLS only as variant selection methods, they can also be used to do the association test for genotype with complex traits. However, both Lasso and SPLS are very computationally intensive. In addition, our analysis is focused on the independent subjects only, which limits our sample size. For future study, it is essential to incorporate family structure that not only increases the size of the sample available for analysis, but also the number of variants. Because SBP and DBP are correlated, it is deficient to analyze them separately. MAP, which is a combination of SBP and DBP, has better power than SBP and DBP separately.

## Competing interests

The authors declare that they have no competing interests.

## Authors' contributions

XFL conducted statistical analyses and drafted the manuscript. JB conceived the study and assisted in drafting the manuscript. All authors read and approved the final manuscript.
